# Genome Sequence of *Corynebacterium pseudotuberculosis* Strain XH02 Isolated from a Boer Goat in Xuanhan, China

**DOI:** 10.1128/genomeA.01329-16

**Published:** 2016-11-23

**Authors:** Zuoyong Zhou, Hexian Li, Mengsi Zhang, Zhiying Wang, Rongqiong Zhou, Shijun Hu, Xiaoxia Li, Xinyue Song, Zheng Zhu

**Affiliations:** Department of Veterinary Medicine, Rongchang Campus of Southwest University, Chongqing, China

## Abstract

We report here the genome sequence of *Corynebacterium pseudotuberculosis* strain XH02, isolated from a Boer goat in China. The genome consists of 2,357,671 bp, with a 52.18% G+C content, 2,263 coding sequences, 21 rRNAs, 49 tRNAs, and 44 predicted pseudogenes.

## GENOME ANNOUNCEMENT

*Corynebacterium pseudotuberculosis*, a pleomorphic, Gram-positive, and facultatively intracellular bacterium, is the etiological agent of caseous lymphadenitis (1). As a zoonotic pathogen, it mainly infects small ruminants, especially sheep and goats (2). It can also infect several other mammalian species, including horses, cattle, pigs, buffaloes, camels, coypus, hedgehogs, as well as humans (3). The diseases caused by *C. pseudotuberculosis* result in great economic loss because the sheep and goat industries are largely affected in Brazil, Australia, United States, South Africa, India, and China (2, 4–8).

With the advantage of high-throughput sequencing, several genome sequences of *C. pseudotuberculosis* are currently available in the NCBI’s database. However, genome sequences of *C. pseudotuberculosis* from indigenous species from China have not been reported, even though more than 30 reports of infection in goats, sheep, and camels in China have been recorded (China National Knowledge Infrastructure database). To better understand and enrich the genomic information of a *C. pseudotuberculosis* isolate from China, we sequenced the genome of *C. pseudotuberculosis* strain XH02.

Strain XH02 was ﬁrst isolated in Xuanhan, China, from a subcutaneous abscess in the neck of a two-year-old Boer goat. A drug sensitivity test showed XH02 to be highly sensitive to 13 antibacterial agents, including chloromycetin, tetracycline, norfloxacin, minocycline, cefoxitin, clarithromycin, roxithromycin, and ceftriaxone, but completely resistant to nitrofurantoin and furazolidone (7).

The genome of *C. pseudotuberculosis* strain XH02 was sequenced on an Illumina HiSeq 4000 platform after the establishment of a qualified Illumina paired-end library. Raw sequence data were generated by the Illumina base-calling software CASAVA version 1.8.2 (http://support.illumina.com/sequencing/sequencing_software/casava.ilmn). The SeqPrep (https://github.com/jstjohn/SeqPrep) and Sickle (https://github.com/najoshi/sickle) software programs were used to remove contamination reads and conduct read trimming with default parameters to obtain clean data. A total of 9,193,824 clean reads were obtained with an average coverage of 928×. The clean data were used for genome assembly with SOAPdenovo version 2.04 (http://soap.genomics.org.cn) with multiple *k*-mer parameters. GapCloser software (http://soap.genomics.org.cn) was subsequently applied to fill up the remaining local inner gaps and correct the single-base polymorphism for the final assembly results. The assembly produced 10 scaffolds with an *N*_50_ value of 549, 021 bp. Both tRNAs and rRNAs were predicted using tRNAscan-SE version 1.23 (9) and RNAmmer version 1.2 (10), respectively.

The genome of *C. pseudotuberculosis* strain XH02 contains 2,357,671 bp with a G+C content of 52.18%, 2,263 coding sequences, 44 pseudogenes, 21 rRNAs, and 49 tRNAs.

### Accession number(s).

The genome sequence obtained with this study was deposited in DDBJ/EMBL/GenBank under the accession number MDWN00000000. The version described in this paper is the first version, MDWN01000000.
